# On the Ageing of High Energy Lithium-Ion Batteries—Comprehensive Electrochemical Diffusivity Studies of Harvested Nickel Manganese Cobalt Electrodes

**DOI:** 10.3390/ma11020176

**Published:** 2018-01-23

**Authors:** Odile Capron, Rahul Gopalakrishnan, Joris Jaguemont, Peter Van Den Bossche, Noshin Omar, Joeri Van Mierlo

**Affiliations:** Mobility Logistics and Automotive Technology Research Centre (MOBI), Department of Electrical Engineering and Energy Technology (ETEC), Vrije Universiteit Brussel (VUB), 1050 Elsene, Belgium; Rahul.Gopalakrishnan@vub.be (R.G.); Joris.Jaguemont@vub.be (J.J.); pvdbos@vub.ac.be (P.V.D.B.); Noshin.Omar@vub.be (N.O.); Joeri.Van.Mierlo@vub.be (J.V.M.)

**Keywords:** Nickel Manganese Cobalt, *Li*^+^ diffusion coefficient, ageing, Cyclic Voltammetry, Galvanostatic Intermittent Titration, Electrochemical Impedance Spectroscopy

## Abstract

This paper examines the impact of the characterisation technique considered for the determination of the $Li^{+}$ solid state diffusion coefficient in uncycled as in cycled Nickel Manganese Cobalt oxide (NMC) electrodes. As major characterisation techniques, Cyclic Voltammetry (CV), Galvanostatic Intermittent Titration Technique (GITT) and Electrochemical Impedance Spectroscopy (EIS) were systematically investigated. $Li^{+}$ diffusion coefficients during the lithiation process of the uncycled and cycled electrodes determined by CV at 3.71 V are shown to be equal to $3.48\times 10^{-10}$ cm${}^{2}$·s${}^{-1}$ and $1.56\times 10^{-10}$ cm${}^{2}$·s${}^{-1}$ , respectively. The dependency of the $Li^{+}$ diffusion with the lithium content in the electrodes is further studied in this paper with GITT and EIS. Diffusion coefficients calculated by GITT and EIS characterisations are shown to be in the range between $1.76\times 10^{-15}$ cm${}^{2}$·s${}^{-1}$ and $4.06\times 10^{-12}$ cm${}^{2}$·s${}^{-1}$, while demonstrating the same decreasing trend with the lithiation process of the electrodes. For both electrode types, diffusion coefficients calculated by CV show greater values compared to those determined by GITT and EIS. With ageing, CV and EIS techniques lead to diffusion coefficients in the electrodes at 3.71 V that are decreasing, in contrast to GITT for which results indicate increasing diffusion coefficient. After long-term cycling, ratios of the diffusion coefficients determined by GITT compared to CV become more significant with an increase about 1 order of magnitude, while no significant variation is seen between the diffusion coefficients calculated from EIS in comparison to CV.

## 1. Introduction

Batteries are now essential components of our daily lives, whether it is in mobile phones, in portable computers, for starting the car engine or for powering the satellite that sends radio communication signals down to earth [1,2,3]. Further, electric vehicles (EVs) including hybrid electric vehicles (HEVs), plug-in hybrid electric vehicles (PHEVs) and pure battery electric vehicles (BEVs) are predicted to dominate the vehicle market [4,5]. Understanding the behaviour of the internal degradation mechanisms occurring due to long term cycling of these batteries and their electrodes is critical [6,7]. Limitations in the lifetime of lithium-ion batteries still reveal often to be an issue in battery applications as in automotive [8,9,10,11]. In recent years, Lithium Nickel Manganese Cobalt (NMC) for use in positive electrodes eventually offered improved cycle life, thermal stability and energy density capabilities for lithium-ion batteries [12,13,14,15]. Generally, charging and discharging capabilities of electrodes are mainly linked to their lithium ion solid phase diffusion coefficient, which is therefore recognised as a significant kinetic characteristic of Lithium ion intercalation electrode materials [16]. In practice, rapid $Li^{+}$ intercalation/deintercalation processes are required by demanding applications such as in EVs. Therefore, electrochemical techniques are widely used and recognised for the determination of $Li^{+}$ diffusion coefficients in liquid electrolytes and in solids [17,18]. However, a lack of knowledge regarding the impact of the characterisation technique and the influence of the ageing on the $Li^{+}$ diffusivity in Nickel Manganese Cobalt (NMC) electrodes still exists. It can be seen in the literature that experimental determinations of the $Li^{+}$ diffusion in the same material can often lead to values differing from several orders of magnitude [19,20,21,22,23,24].

Therefore, a comprehensive diffusivity study for the intercalation process of $Li^{+}$ in uncycled and cycled NMC electrodes is presented with this paper. Three major electrochemical characterisation techniques: Cyclic Voltammetry (CV), Galvanostatic Intermittent Titration Technique (GITT) and Electrochemical Impedance Spectroscopy (EIS) were investigated. In this work, particularly the last two GITT and EIS techniques were combined and performed as one single test protocol. Using these techniques, the impact of the characterisation technique on the determination of $Li^{+}$ solid state diffusion coefficients in uncycled and cycled electrodes and their dependency on the lithium content are demonstrated.

## 2. Materials and Methods

**Figure 1:**
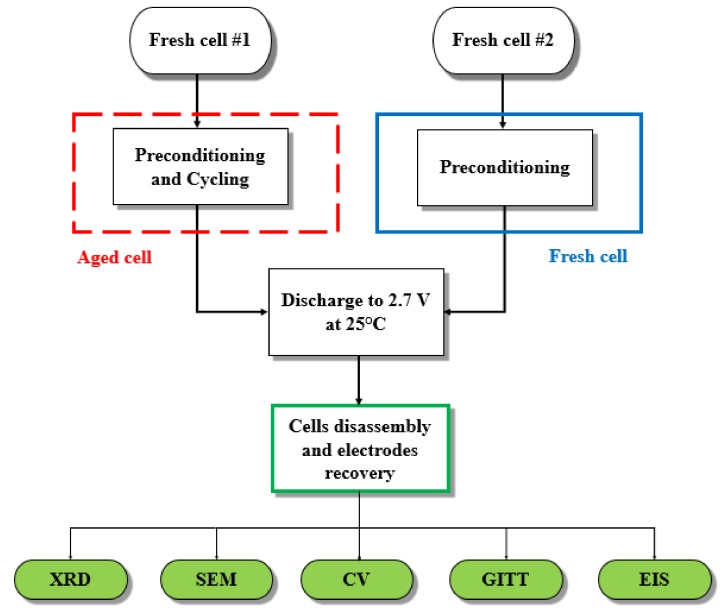
Flow diagram of battery cells and electrodes experimental procedures.

A commercial high energy pouch cell type of 20 Ah nonimal capacity (manufactured by EIG) is studied in this work. The cell incorporates $Li\left(Ni_{0.4}Mn_{0.4}Co_{0.2}\right)O_{2}$ (NMC) and graphite materials for the positive and the negative electrodes, respectively. The flow diagram of the experimental investigations carried out on battery cells and electrodes is presented in Figure 1.

According to the different samples of battery cells and electrodes listed in Table 1, a first battery cell was cycled at 35 ${}^{\circ }$C temperature inside a CTS climate chamber [25]. Long-term charge/discharge cycling was carried out by steps of 100 cycles with an PEC [26] (Leuven, Belgium) ACT battery tester. The cell was charged/discharged in the voltage range [3 V; 4.15 V] with a current rate equal to 1 C (20 A). Discharging the cell was achieved by using constant current. For its charge, a constant current was applied until its upper cut off voltage is reached, followed by a constant voltage charge for the current to drop below 0.05 C (1 A). Next to this cell, a second cell that will be used as reference is taken to be dismantled without being cycled for the analysis of its electrodes.

For electrochemical characterisation of its electrodes, the cycled battery cell was discharged at a current rate equivalent to C/100 (0.2 A) to reach 2.7 V. Electrodes sheets were taken out from the battery cell and cleaned with dimethyl carbonate (DMC) for removing the electrolyte. Samples were punched from the recovered electrodes sheets and stored inside glass bottles covered on top with aluminium foil. Thereafter, electrode samples were dried inside a glass vacuum chamber for 12 h at 120 ${}^{\circ }$C. EL-CELLs [27] (Hamburg, Germany) electrochemical equipments were used for testing of 18 mm diameter electrode samples. A Lithium foil was used as counter electrode, which was separated by a 24 microns thickness and 24 mm diameter Celgard^®^ polypropylene (PP) separator sheet. Enough to wet the electrode, 0.2 mL of Sigma-Aldrich^®^ EC:DMC (1:1 *w*/*w*) electrolyte (1 mol/L solution of lithium hexafluorophosphate ($LiPF_{6}$) in a mixture of ethylene carbonate and dimethyl carbonate) was filled in the EL cells. In addition to the cycled battery cell, a non-cycled battery cell (or reference) was disassembled following the same procedure.

The characterisation of both uncycled and cycled positive NMC electrodes was conducted with a set of five different techniques as follows: X-ray Diffraction (XRD), Scanning Electron Microscopy (SEM), Cyclic Voltammetry (CV), Galvanostatic Intermittent Titration Technique (GITT), Electrochemical Impedance Spectroscopy (EIS).

Ex-situ X-ray diffraction characterisation has been performed for the determination of the maximum lithium ion concentration in the solid phase of the electrodes which is required in the determination of the $Li^{+}$ solid phase diffusion by EIS. Electrode samples were analysed with a Bruker (Karlsruhe, Germany) D5000 X-ray diffractometer with RX Cuα source. For the experiments, the 2θ angle was set to vary between 10 and 80 degrees. The experimental XRD patterns were analysed by Rietveld refinements. For each electrode, one fully lithiated (corresponding to 3 V) and one fully delithiated (corresponding to 4.15 V) samples were prepared.

Scanning electron microscopy has been carried out for the analysis of the morphological features associated to the microstructure of the uncycled and cycled NMC electrodes with a JEOL (Tokyo, Japan) JSM-IT300 scanning electron microscope under 15 kV accelerating voltage. In this work, morphological studies carried out by SEM are aimed for the determination of the active material particles radii which are involved in the determination of the $Li^{+}$ solid phase diffusion by GITT.

Cyclic voltammetry experiments were conducted on the uncycled and cycled electrodes with scan rates between 0.011 mV·s${}^{-1}$ and 0.319 mV·s${}^{-1}$. Three repetitions of the potential sweeps for each scan rate were carried out. The Galvanostatic Intermittent Titration Technique has been performed together with the Electrochemical Impedance Spectroscopy characterisation in one test protocol. Both techniques have been carried out and designed for the determination of the $Li^{+}$ diffusion coefficient in the uncycled and cycled NMC electrodes as function of their lithium content. The electrodes were charged to their upper cut-off potential using a CC-CV method (C/30-0.5 C) followed at each 10% SoC by a sequence of constant discharge current steps and relaxation periods (*I* = 0 A) for the stabilization of the potential before to proceed to EIS measurements. The sinusoidal excitation signal and the frequency ranges associated to the electrochemical impedance spectroscopy measurements were equal to 3 mV and [1 MHz; 10 mHz] respectively. The excitation voltage was optimised to reduce the noise level during the measurements and still satisfy the linearity condition intrinsic to this technique. All experiments were performed with a Bio-Logic (Claix, France) VSP electrochemical workstation and by keeping the electrodes at 25 ${}^{\circ }$C in a CTS (Hechingen, Germany) type T climate chamber.

## 3. Results and Discussion

### 3.1. Long-Term Cycling

**Figure 2:**
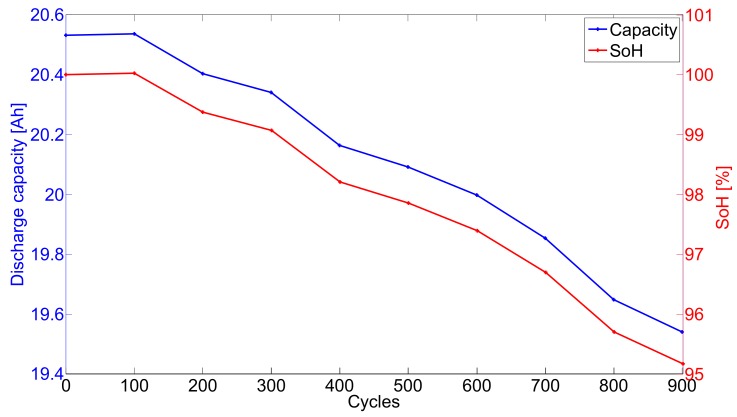
Aged battery cell discharge capacity and SoH evolutions with the number of cycles.

The evolution of the discharge capacity and the State of Health (SoH) during long-term cycling of the cell are illustrated in Figure 2.

Initially the cell presented a capacity slightly higher (2%) than specified by the manufacturer. After 100 cycles at 35 ${}^{\circ }$C , no capacity drop is observed. Even a slight increase can be noticed. This is possibly due to the 35 ${}^{\circ }$C temperature condition which leads to enhanced electrochemical kinetics and prevent from performances degradations in the early stage of the long-term cycling. Beyond 100 cycles, it is observed that capacity fade occurs during cycling of the cell. On the long-term, 35 ${}^{\circ }$C temperature also enhance undesired electrochemical side reactions, resulting in performances degradations and capacity fade. After 900 cycles the capacity was dropped to 95% of the initial capacity and the cell was reserved for post-mortem analysis.

### 3.2. Material Identification

#### 3.2.1. Ex-Situ X-ray Diffraction

**Figure 3:**
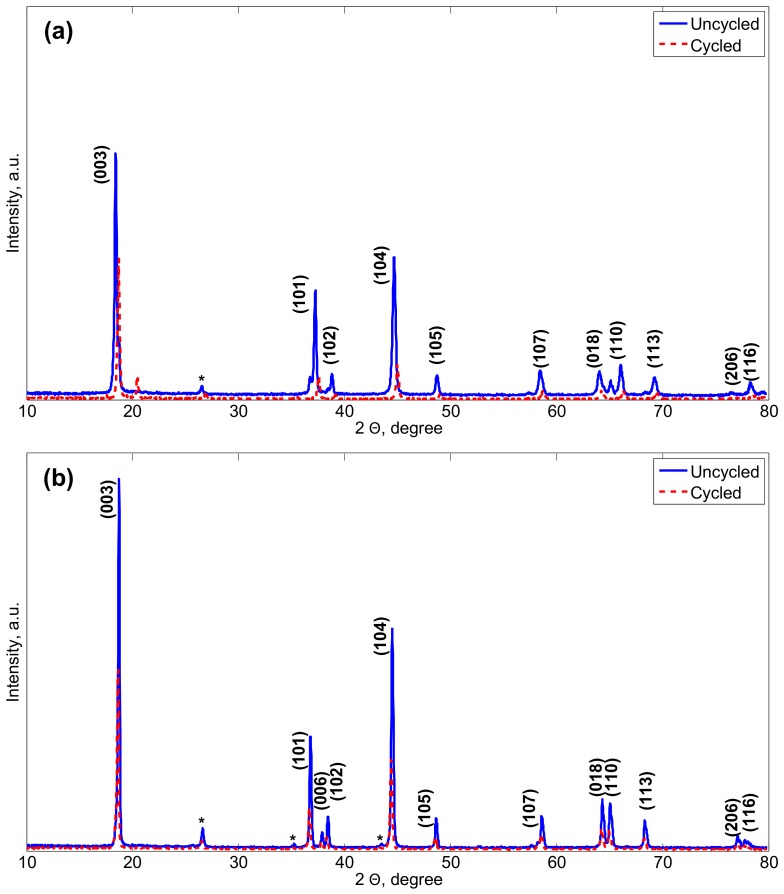
XRD patterns of the uncycled and cycled NMC electrodes in (**a**) their lithiated states and (**b**) delithiated states.

Figure 3 illustrates the XRD patterns of the uncycled and cycled NMC electrodes in their lithiated and delithiated states. The characteristic peaks are corresponding to the trigonal (or rhombohedral) α-$NaFeO_{2}$ (R-3m) structure as reported in [28,29]. The separation of the doublets peaks (006)/(102) and (108)/(110) reveals highly ordered layered structures with a good hexagonal ordering for both electrodes. After long-term cycling, the diffraction peaks of the cycled electrode are reduced compared to those of the uncycled electrode. A new peak is observed in Figure 3a around 20${}^{\circ }$ angle which translates for the formation of an amorphous phase. This might indicate for side reactions between the electrodes and the electrolyte and further for the degradation of their crystal structure. Particularly, the amorphous phase is believed to present a low conductivity and hinder the $Li^{+}$ insertion/extraction process [30].

Experimental XRD patterns were recorded and the crystal structures of the electrodes matching their peaks were determined from Rietveld refinements. Changes in the lattice parameters are generally tracked for understanding modifications induced in the electrodes crystal structure after cycling. Corresponding *a* and *c* lattice parameters and unit cells volumes are listed in Table 2. Lattice parameters *a* and *c* are related to the average metal-metal intra slab distance and to the average metal-metal inter slab distance, respectively. Considering the rhombohedral crystal structure of the electrodes for which the volume of a unit cell equals to one third of that of an hexagonal unit cell, the maximum $Li^{+}$ concentration $C_{s,max}$ in the electrodes is computed as follows with Equation (1):

(1) $$C_{s,max}=\frac{1}{\left(V/3\right)\times \left(N_{A}\right)}$$

where V is the volume corresponding to one hexagonal unit cell and $N_{A}$ is the Avogadro constant ($6.02\times 10^{23}$ mol${}^{-1}$).

Table 2 summarises the lattice parameters calculated from Rietveld refinements and the calculated maximum $Li^{+}$ concentrations in the lithiated and delithiated electrodes.

Difference in the structural parameters is seen between the lithiated and delithiated states of both uncycled and cycled electrodes. This indicates as expected the dependence of the unit cell parameters on the lithium content in the electrodes. Increase in the lattice parameter *a* and decrease in the lattice parameter *c* are observed to occur with the $Li^{+}$ insertion process in both electrodes types. The unit cell volume evaluated for the lithiated electrodes are seen to be greater than that in the delithiated electrodes. Consequently, maximum $Li^{+}$ concentrations in the lithiated electrodes are then well lower than in the delithiated electrodes. These observations are in agreement with the literature [31]. Further, uncycled and cycled electrodes are seen to also differ from their unit cell parameters. Cycling is observed to induce shrinkage in the unit cells volume and the lattice parameter *c* of the lithiated electrode in comparison to the initially uncycled electrode. This reflects the loss of atoms from the crystal structure of the lithiated electrodes. Shifts in the peaks positions towards higher 2θ angles is observed after cycling for the lithiated electrodes, while characteristic peaks of the delithiated electrodes are noticed to move with ageing towards lower 2θ angles. In practice, shifts towards lower angles translate for vacancies in the crystal structure of the electrodes while shifts to higher 2θ are associated to the existence of ions with an ionic radius lower than $Ni^{2+}$.

Results from the analysis of the crystal structure parameters with ageing and the lithiation states of the NMC electrodes are presented in Table 3.

It can be observed that the c/a ratio for both uncycled and cycled NMC electrodes is greater than the 4.899, which demonstrates their crystalline layered structure [32]. The I(003)/I(104) intensity ratio is inversely proportional to the degree of cation mixing between $Li^{+}$ and $Ni^{2+}$ in the lithium layer [33]. Cation mixing is a structural disorder where a partial occupation of $Ni^{2+}$ on the crystallographic 3b sites of a $Li^{+}$ and conversely a partial occupation of $Li^{+}$ in 3a sites of $Ni^{2+}$ in transition metal layers may occur considering the close ionic radii of $Li^{+}$ (0.76 Å) and $Ni^{2+}$ (0.69 Å). In practice, this phenomenon is known for deteriorating the electrochemical performance of the layered oxides materials by decreasing their discharge capacity and hindering the diffusion pathway of $Li^{+}$ [34,35]. A higher concentration of cation mixing would lead to materials that are less ordered with a greater $Li^{+}$ diffusion barrier [36]. I(003)/I(104) intensity ratios greater than 1.2 indicate little cation mixing for both uncycled and cycled electrodes [37]. The (I(006)+I(102))/I(101) ratio (or so called R-factor) informs about the hexagonal ordering of the crystal structure of the electrodes. In practice, a small ratio translates into a good separation in the transition metal and the $Li^{+}$ respective planes [38,39] yielding to a good hexagonal ordering [40]. With a higher R factor after cycling, it is found that the hexagonal ordering of the initially delithiated electrodes is affected by ageing.

#### 3.2.2. Scanning Electron Microscopy

**Figure 4:**
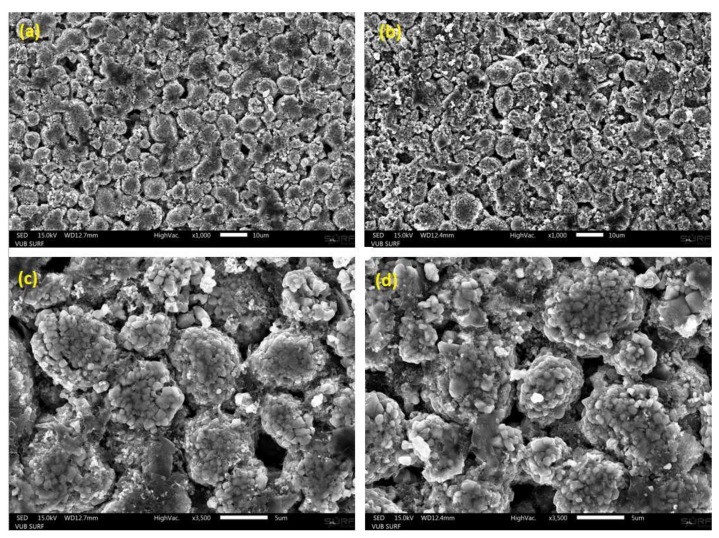
Scanning electron microscopy (SEM) micrograph of the uncycled and cycled NMC electrodes at ×1000 magnification rate (**a**,**b**) and ×3500 magnification rate (**c**,**d**).

Figure 4 illustrates the SEM images of the uncycled (a,c) and cycled (b,d) NMC electrodes at different magnifications. The morphology of the active material particles of the electrodes were examined from powders in their delithiated state. Both uncycled and cycled NMC active material particles are seen to present spherical morphology. A slightly increased brightness is shown by the SEM images of the cycled compared to the uncycled electrodes. This would suggest a relative higher amount of non-electronic conductive material in the cycled electrodes.

**Figure 5:**
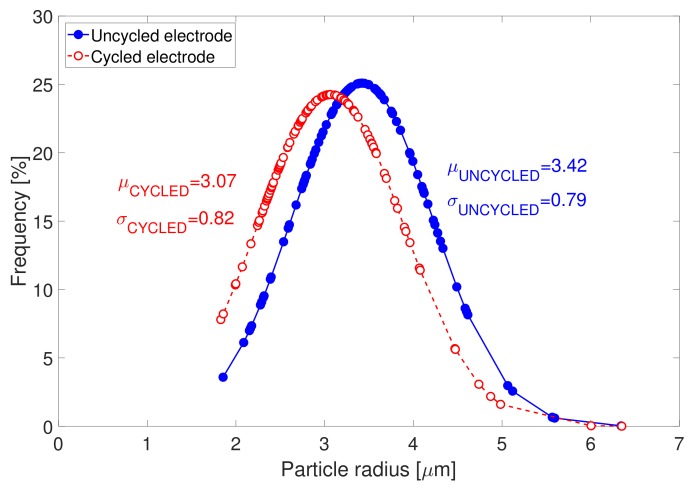
Particle size distribution of uncycled and cycled NMC electrodes.

The determination of the radius of the primary particles and the secondary particles (or also designated by “agglomerates”) were conducted from the determination of the particle size distribution of the SEM images in Figure 4. Particle size distributions associated to both electrodes types are displayed in Figure 5. After cycling, particle size distribution is seen to shift towards lower particle radii. The radius of the secondary particles in the uncycled electrodes is identified to be equal to 3.42 μm. After cycling, the agglomerates are observed to have slightly decomposed into smaller particles of 3.07 μm radius. This might possibly indicate for the starting of chemical ex-foliation/corrosion and particle cracking taking place in the cycled electrode samples due to cycling. The distribution of the cycled particles radii is also seen to be broader than that of the uncycled electrode, which suggests for more heterogeneous particle size distribution in cycled electrode in comparison to uncycled electrode.

### 3.3. Electrochemical Characterisation

#### 3.3.1. Cyclic Voltammetry

**Figure 6:**
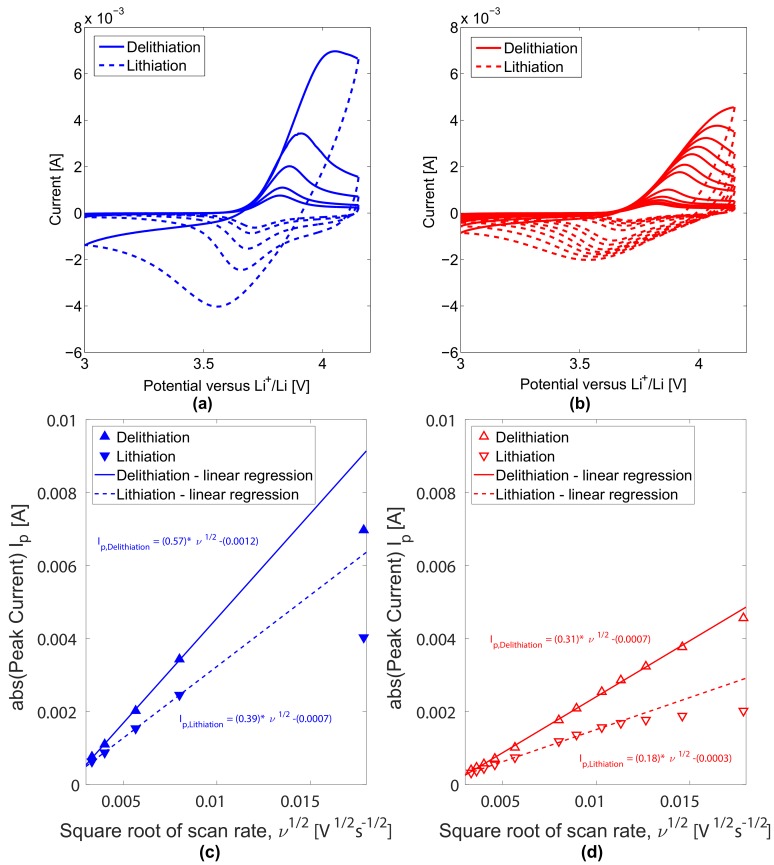
Cyclic voltammograms and redox peak currents as a function of the square root of the scan rate for uncycled and cycled positive electrodes. (**a**) Cyclic voltammograms at scan rates 0.011 mV·s${}^{-1}$, 0.016 mV·s${}^{-1}$, 0.032 mV·s${}^{-1}$, 0.064 mV·s${}^{-1}$, 0.319 mV·s${}^{-1}$ for uncycled NMC electrodes. (**b**) Cyclic voltammograms at scan rates 0.011 mV·s${}^{-1}$, 0.013 mV·s${}^{-1}$, 0.016 mV·s${}^{-1}$, 0.021 mV·s${}^{-1}$, 0.032 mV·s${}^{-1}$, 0.064 mV·s${}^{-1}$, 0.080 mV·s${}^{-1}$, 0.106 mV·s${}^{-1}$, 0.128 mV·s${}^{-1}$, 0.160 mV·s${}^{-1}$, 0.213 mV·s${}^{-1}$, 0.319 mV·s${}^{-1}$ for cycled NMC positive electrodes; Redox peak currents as a function of the square root of the scan rate for uncycled (**c**) and cycled (**d**) positive electrodes.

Cyclic voltammograms related to the uncycled and cycled electrodes at different scan rates between 0.011 mV·s${}^{-1}$ and 0.319 mV·s${}^{-1}$ in the potential range [3 V; 4.15 V] are shown in Figure 6a,b, respectively. Voltammograms of both electrodes types exhibit a single couple of redox peaks corresponding to the oxidation and reduction of $Ni^{2+}/Ni^{4+}$ [32]. Oxidation peaks during the delithiation process of the uncycled and cycled electrodes are observed at 3.8211 V and 3.8213 V, respectively. During the lithiation process of the uncycled and cycled electrodes reduction peaks are observed at 3.7157 V and 3.7107 V, respectively. At the same scan rate, after cycling, the anodic peak potential is seen to slightly shift towards higher values while the cathodic peak potential tends to slightly shift towards lower values. The difference between the redox potentials becomes then slightly larger. Cycled electrode is found to present slightly less symmetrical voltammograms resulting from increased potential difference $\Delta \phi_{p}$ (in Table 4) and higher polarization degree. This observation suggests for a lower reversibility during the lithiation/delithiation processes of the cycled electrodes due to ageing. Accordingly, long-term cycling affects the $Ni^{2+}/Ni^{4+}$ redox reaction and the polarisation of the electrochemical oxidation/reduction reactions in the electrodes.

As shown from the fitting results in Figure 6c,d, redox peaks currents of both electrodes exhibit a linear relationship with the square root of the scan rate below 0.128 mV·s${}^{-1}$ scan rate. The linear relationship demonstrates that the redox processes of both electrodes is rate determined by the $Li^{+}$ diffusion in the electrodes for scan rates below the critical scan rate of 0.128 mV·s${}^{-1}$ . For both electrodes, linear fitting of anodic peak currents are seen to display higher slopes than that of the cathodic peaks. Further, fittings of the redox peak currents for the cycled electrode exhibit lower slopes. Both scan rate and cycling are affecting the reversibility of the NMC electrodes.

Diffusion coefficients are calculated from the slope of the plot of the peak currents versus the square root of the scan rates as in Equation (2), which applies for diffusion-controlled reactions with the assumption of semi-infinite diffusion [41]

(2) $$i_{p}=2.69\times 10^{5}n^{\left(3/2\right)}AD^{\left(1/2\right)}C^{0}\nu^{\left(1/2\right)}$$

where $i_{p}$ is the peak current, *n* is the number of electrons transfered, *D* is the diffusion coefficient, *C* is the $Li^{+}$ concentration, *A* is the electrode surface area and ν is the scan rate.

The anodic and cathodic peak potentials, $\phi_{pa}$ and $\phi_{pc}$ in Figure 6a,b and their potential difference $\Delta \phi_{p}$ are presented in Table 4. $Li^{+}$ solid phase diffusion coefficients $D_{s,pa}$ and $D_{s,pc}$ in the electrodes at the corresponding redox potentials are calculated in Table 4, to be in the range between $1.56\times 10^{-10}$ cm${}^{2}$·s${}^{-1}$ and $5.10\times 10^{-10}$ cm${}^{2}$·s${}^{-1}$ and corresponding values for mean and standard deviation are presented in Table 5. In both electrodes, the diffusion of $Li^{+}$ is around 1.5 times faster during the delithiation compared to the lithiation process. This observation correlates with $Li^{+}$ diffusion coefficients determined by cyclic voltammetry characterisation of electrodes in lithium-ion batteries [42,43,44]. Ratios in diffusion coefficients between uncycled and cycled electrodes indicate the $Li^{+}$ diffusion coefficient to decrease with ageing. It can be seen that long term cycling induced a decrease in the $Li^{+}$ diffusion about half of the diffusion initially associated to the uncycled electrode. These lower $Li^{+}$ diffusion coefficients indicate for worsen $Li^{+}$ intercalation/deintercalation capabilities and for lessen reversibility of these processes in the cycled electrodes.

#### 3.3.2. Galvanostatic Intermittent Titration Technique

Galvanostatic Intermittent Titration Technique consists in applying constant current pulses during a given time and measuring the potential response of the studied system. Fundamentally, chemical diffusion coefficient, *D* in a system can be determined by GITT with Equation (3) (as long as $t<<\frac{r^{2}}{D}$) [45,46] in case particles can be considered as semi-infinite solids:

(3) $$D=\frac{4}{\pi }{\left(\frac{1}{SFz_{A}}\right)}^{2}{\left(\frac{I_{0}\left(dE/dc\right)}{dE/d\sqrt{t}}\right)}^{2}$$

with *S*, *F*, $z_{A}$ representing the particle area involved in the current pulses, the Faraday constant, the charge number of the ion and $I_{0}$, *E*, *c* designate the galvanostatic current step, the measured potential and the surface concentration, respectively.

Basically, determination of diffusion coefficient with GITT characterisation rely on several assumptions. For $Li^{+}$ diffusion coefficient in electrodes, one dimensional semi-infinite particles are considered. Second, the transport of lithium is assumed to follow the Fick’s law. Third, diffusion coefficient are assumed not to vary during the current pulses. For small and short current pulses, dE/dc and $dE/d\sqrt{t}$ derivatives can be considered as constant and approached by $\Delta E_{s}/\Delta c$ and $\Delta E_{\tau }/\sqrt{t}$. Considering the relations between *S*, *F*, $z_{A}$ and $I_{0}$, $Li^{+}$ diffusion coefficient can be extracted from the data associated to each current pulse according to Equation (4) [45,46] (as long as $\tau <<\frac{L^{2}}{D_{Li^{+}}}$):

(4) $$D_{Li^{+}}=\frac{4L^{2}}{\pi \tau }{\left(\frac{\Delta E_{s}}{\Delta E_{\tau }}\right)}^{2}$$

where *L* is the characteristic diffusion length equal to $R_{s}/3$ for spherical-like active material particles with a radius $R_{s}$ determined in Section 3.2.2, τ is the duration of the discharge pulses, $\Delta E_{s}$ and $\Delta E_{\tau }$ represent the changes in the steady state potential and in the transient potential of the electrode. In this paper, GITT combined with EIS measurements were conducted together on the electrodes in a single test protocol. The GITT characterisation was defined by discharge current pulses of amplitude equivalent to C/30 i.e., 195 μA (uncycled electrode) and 186 μA (cycled electrode). The duration of each pulse corresponds to the time needed to reach 10% discharge capacity of the electrodes. A total of 10 discharge current steps were performed on both uncycled and cycled electrodes. Each current pulse was followed by a relaxation period with a duration equal to reach less than 0.1 mV/h potential variation. The proposed methodology allows to reach a very stable potential (less than 0.1 mV/h potential variation) at the level for which the EIS measurements are performed. Further the reduction of the total test time compared to the case where the two experiments are performed separately is also an advantage of this methodology.

**Figure 7:**
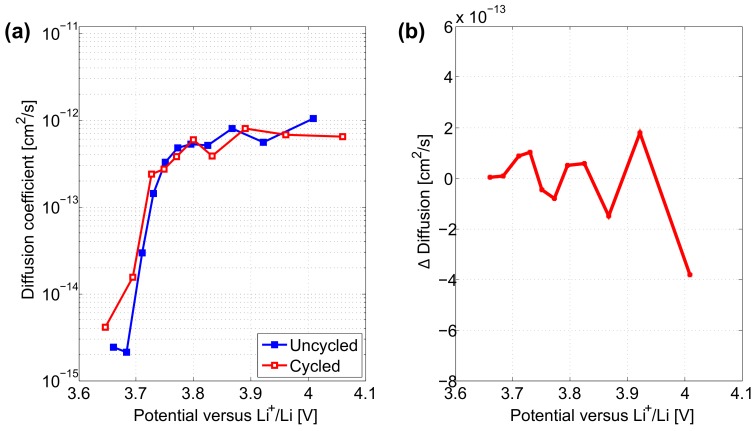
(**a**) Diffusion coefficient in uncycled and cycled positive electrodes determined by GITT characterisation. (**b**) Difference between cycled and uncycled electrodes.

Figure 7a shows the evolution of the diffusion coefficient in the solid phase of both electrodes as a function of the potential. From the start of the discharge down to 3.8 V, $Li^{+}$ diffusion coefficients in both electrodes remain between $3\times 10^{-13}$ cm${}^{2}$·s${}^{-1}$ and $1\times 10^{-12}$ cm${}^{2}$·s${}^{-1}$. This reflects a rather law kinetic barrier for the lithiation of the electrodes. Further below 3.8 V, diffusion coefficients in the uncycled and cycled electrodes are decreasing promptly towards $10^{-15}$ cm${}^{2}$·s${}^{-1}$. At the end of the lithiation process most active material particles are fully lithiated and the insertion of additional lithium in remaining delithiated particles becomes more difficult. In comparison to the uncycled electrode, the cycled electrode shows slightly lower $Li^{+}$ diffusion in the beginning of the discharge. This lower diffusion coefficient reflects a decreased conductivity properties compared to that of the initially uncycled electrode. Although from 3.71 V, cycled electrode tends to exhibit relatively higher $Li^{+}$ diffusion coefficient in the end of the lithiation process compared to the initially uncycled electrode.

Diffusion coefficients in cycled and uncycled electrodes determined by GITT at 3.71 V and their ratio are presented in Table 6. After long-term cycling, electrodes exhibit a diffusion coefficient equal to $8.60\times 10^{-14}$ cm${}^{2}$·s${}^{-1}$ which is equivalent to an increase of almost 3 times compared to initially uncycled electrodes. In contrast to CV, $Li^{+}$ diffusion coefficient determined at 3.71 V by GITT is seen to increase with ageing.

#### 3.3.3. Electrochemical Impedance Spectroscopy

Electrochemical Impedance Spectroscopy was conducted together with GITT characterisation technique. Basically, diffusion phenomenon is associated to an impedance, namely the Warburg impedance. At high frequencies the diffusion path for the species to diffuse is rather limited, hence the Warburg impedance is low. At low frequencies the Warburg impedance is increased considering an increase diffusion path for the diffusing species. Warburg impedance is defined as in Equation (5):

(5) $$Z_{W}=\sigma {\left(\omega \right)}^{-1/2}\left(1-j\right)$$

which assumes the diffusion layer to be of infinite thickness. Warburg coefficient σ, associated to the diffusion tail in the impedance spectra of the species reactants is defined in Equation (5) [17] as:

(6) $$\sigma =\frac{RT}{n^{2}F^{2}A\sqrt{2}}\left(\frac{1}{C_{O}\sqrt{D_{O}}}+\frac{1}{C_{R}\sqrt{D_{R}}}\right)$$

where ω stands for the radial frequency, $D_{O}$ and $D_{R}$ represent the diffusion coefficient of the oxidant and the reductant, $C_{O}$ and $C_{R}$ represent the concentration of the oxidant and the reductant, *R* is the gas constant constant, *T* is the absolute temperature, *A* is the surface area of the electrode, *n* is the number of electrons transferred in the oxidation or reduction electrochemical reaction, *F* is the Faraday constant. Particularly Equation (7) applies for reversible reaction, controlled by the diffusion of single species due to a concentration gradient as in the case of $Li^{+}$ diffusion phenomenon:

(7) $$D_{Li^{+}}=\frac{R^{2}T^{2}}{2A^{2}n^{4}F^{4}C_{Li^{+}}^{2}\sigma^{2}}$$

with $C_{Li}$ is the concentration of lithium ion in the solid phase of the electrode.

**Figure 8:**
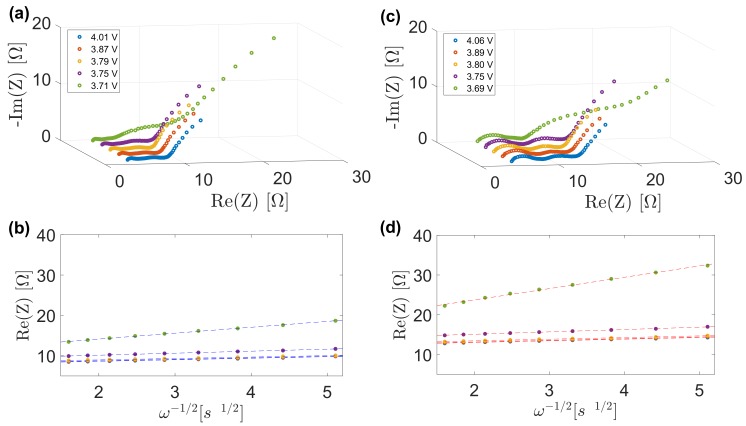
Samples of impedance spectra and corresponding linear fits for uncycled (**a**,**b**) and cycled (**c**,**d**) electrodes.

An electrical equivalent circuit including three resistances accounting for electrolyte or solution resistance, the surface film and the charge transfer resistances, as well as two constant phase elements for the surface film and the double layer capacitances, is associated to the impedance spectra of both uncycled and cycled electrodes in Figure 8. Hence, Warburg factor, σ in this paper is determined by Equation (8):

(8) $$Z_{re}=R_{s}+R_{sei}+R_{ct}+\sigma {\left(\omega \right)}^{-1/2}$$

where $Z_{re}$ is the real part of the electrodes impedance, ω is the angular frequency, σ is determined by linear fitting of the $Z_{re}$ versus ${\left(\omega \right)}^{-1/2}$ plot in Figure 8.

**Figure 9:**
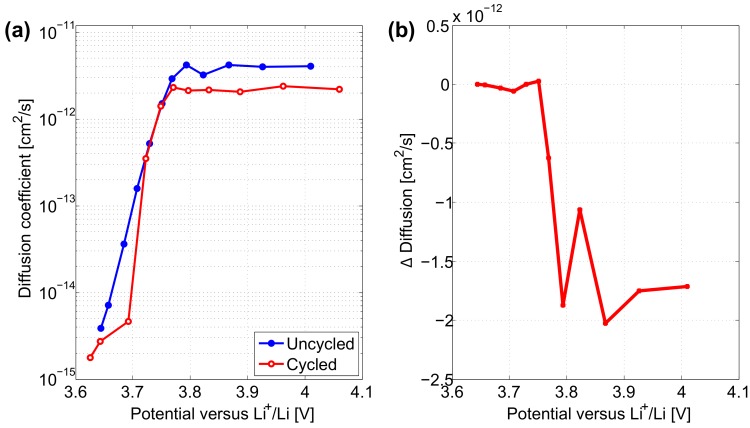
(**a**) Diffusion coefficient in uncycled and cycled positive electrodes determined by EIS characterisation. (**b**) Difference between cycled and uncycled electrodes.

In Figure 9a during the lithiation process, the cycled electrode exhibits lower or equal diffusivity in comparison to the uncycled electrode. The difference between the $Li^{+}$ diffusion in uncycled and cycled electrodes in the potential range [3.75 V; 4.15 V] is more pronounced compared to that determined by GITT in Figure 7a.

Diffusion coefficients in cycled and uncycled electrodes determined by EIS at 3.71 V and their ratio are presented in Table 7. After long-term cycling, electrodes exhibit a diffusion coefficient equal to $7.14\times 10^{-14}$ cm${}^{2}$·s${}^{-1}$ which is equivalent to about half of the diffusion value in initially uncycled electrodes. Similarly to CV, $Li^{+}$ diffusion coefficients determined at 3.71 V by EIS are seen to decrease with ageing.

#### 3.3.4. Comparison of the Techniques

**Figure 10:**
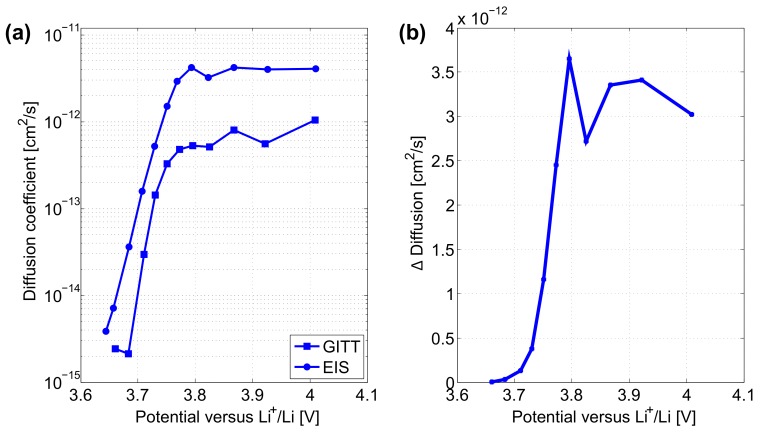
(**a**) Diffusion coefficients in uncycled positive electrodes from GITT and EIS characterisations. (**b**) Difference between the two characterisation techniques.

Further comparison of the three techniques is presented in this section for both uncycled and cycled electrodes. Figure 10a illustrates the solid phase diffusion coefficients obtained by GITT and EIS characterisations on uncycled electrodes. Both techniques indicate the same decreasing trend for the solid phase diffusion coefficient in the electrodes during their lithiation process. Overall, disparities in lithium ion diffusion coefficients in the electrodes from both techniques are found below $4\times 10^{-12}$ cm${}^{2}$·s${}^{-1}$ in Figure 10b. EIS shows higher diffusion coefficients compared to that calculated from GITT. Differences in $Li^{+}$ diffusion coefficients determined by GITT and EIS characterisations might originate from inhomogeneities intrinsic to the electrodes material. Disparities that are typically observed in practice with regard to the shape and size of the electrodes active material particles, may affect the diffusion time constant associated to both techniques and therefrom lead to disparities in their diffusion coefficient results [47].

**Figure 11:**
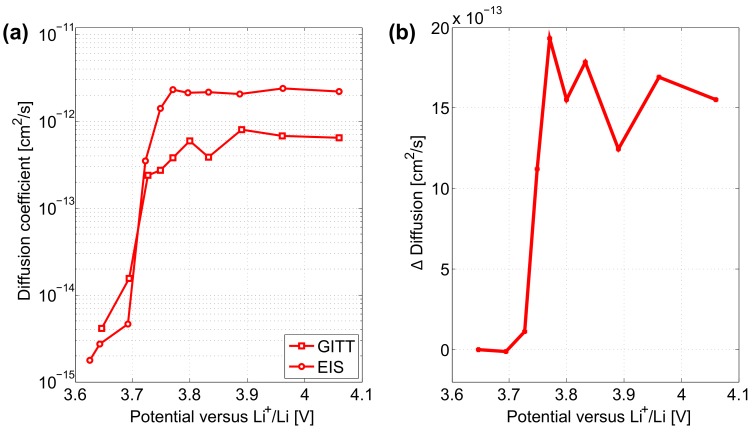
(**a**) Diffusion coefficients in cycled electrodes from GITT and EIS characterisations. (**b**) Difference between the two characterisation techniques.

Solid phase diffusion coefficients obtained from GITT and EIS characterisations on cycled electrode are compared in Figure 11a. As for the uncycled electrode, both GITT and EIS characterisations for the determination of the diffusion coefficient in the cycled electrodes show the same decreasing trend throughout the insertion of the $Li^{+}$ in the electrodes. Two regions can be identified from the electrode potential equal to 3.71 V, above which EIS leads to greater diffusion than GITT and reversely to lower diffusion below. Disparities in the diffusion coefficient values from both techniques are observed to be below $2\times 10^{-12}$ cm${}^{2}$·s${}^{-1}$ in Figure 11b.

**Figure 12:**
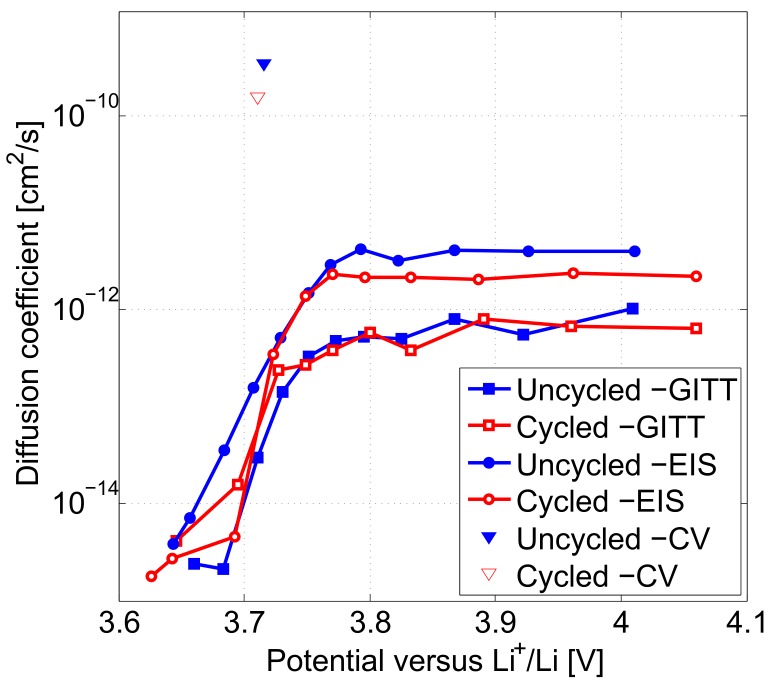
Diffusion coefficients in both electrode types from GITT, EIS and CV characterisations.

Figure 12 presents the comparison of the $Li^{+}$ solid phase diffusion coefficients calculated by GITT, EIS and CV characterisations on uncycled and cycled electrodes. All three techniques agree on $Li^{+}$ diffusion coefficient in the electrodes to vary in the range between $1.76\times 10^{-15}$ cm${}^{2}$·s${}^{-1}$ and $3.48\times 10^{-10}$ cm${}^{2}$·s${}^{-1}$. In Figure 12, in contrast to CV and EIS techniques, diffusion coefficient in the electrodes at 3.71 V determined by GITT is shown to increase with ageing.

Table 8 lists the ratios of the diffusion coefficients determined for both electrodes by GITT and EIS techniques compared to CV. These ratios are ranging between $8.56\times 10^{-5}$ and $5.54\times 10^{-4}$.

For uncycled and cycled electrodes, diffusion coefficients calculated from CV show greater values compared to those determined by GITT and EIS. Diffusion coefficients determined by combined GITT and EIS experimental protocol are associated to a longer time domain and hence closer from the equilibrium state. After long term cycling, ratios of the diffusion coefficients from GITT compared to CV become more significant with an increase about 1 order of magnitude, while no significant variation due to ageing is seen between the diffusion coefficients from EIS in comparison to CV.

## 4. Conclusions

With this paper, the impact of the characterisation technique considered for the determination of the $Li^{+}$ solid state diffusion coefficient in uncycled as in cycled Nickel Manganese Cobalt oxide (NMC) electrodes was systematically investigated by Cyclic Voltammetry, Galvanostatic Intermittent Titration Technique and Electrochemical Impedance Spectroscopy. $Li^{+}$ diffusion coefficients in the uncycled and cycled electrodes determined by CV at 3.71 V are found equal to $3.48\times 10^{-10}$ cm${}^{2}$·s${}^{-1}$ and $1.56\times 10^{-10}$ cm${}^{2}$·s${}^{-1}$, respectively. Diffusion coefficients calculated from GITT and EIS demonstrate the same decreasing trend throughout the lithiation process of the electrodes and are ranging between $1.76\times 10^{-15}$ cm${}^{2}$·s${}^{-1}$ and $4.06\times 10^{-12}$ cm${}^{2}$·s${}^{-1}$. Results presented in this paper suggest that CV lead to greater diffusion coefficient values compared to those determined by GITT and EIS. At 3.71 V potential, CV and EIS techniques lead to diffusion coefficients that are decreasing with ageing, in contrast to GITT. After long-term cycling, an increase about 1 order of magnitude is found in the ratios of the diffusion coefficients from GITT compared to CV, while no significant variation due to ageing is seen between the diffusion coefficients from EIS in comparison to CV.

## Figures and Tables

**Table 1:** Battery cells/electrodes and corresponding experimental testings.

Full Cells/Electrodes	Fresh/Uncycled	Aged/Cycled
**Full cells**	5 charge/discharge cycles (0.3 C/0.3 C) at 25 ${}^{\circ }$C (Preconditioning).	900 charge/discharge cycles (1 C/1 C) at 35 ${}^{\circ }$C (Accelerated Ageing).
	Discharge at 25 ${}^{\circ }$C to 2.7 V for disassembly and characterisation of electrodes.	
**Electrodes**	XRD, SEM, CV, GITT, EIS.	

**Table 2:** Variations of the crystal structure parameters of the NMC electrodes with their lithiation state and ageing.

	a-Axis (Å)		c-Axis (Å)		V (${Å}^{3}$)		$C_{s,max}$ (mol/m${}^{3}$)	
Electrode State	Uncycled	Cycled	Uncycled	Cycled	Uncycled	Cycled	Uncycled	Cycled
Lithiated	2.868	2.869	14.274	14.266	101.712	101.699	48,995	49,001
Delithiated	2.830	2.827	14.452	14.480	100.261	100.266	49,704	49,702

**Table 3:** Variations of the crystal structure parameters of the electrodes with their lithiation state and ageing.

	NMC			
	Delithiated		Lithiated	
Parameter	Uncycled	Cycled	Uncycled	Cycled
c/a	5.10	5.12	4.98	4.97
I(003)/I(104)	1.67	1.97	1.73	4.08
(I(006)+I(102))/I(101)	0.46	0.52	0.33	0.17

**Table 4:** Redox peaks characteristics of NMC uncycled and cycled electrodes.

Electrode	$\phi_{pa}\left[V\right]$	$\phi_{pc}\left[V\right]$	$\Delta \phi_{p}\left[V\right]$	$D_{s,pa}$ [cm${}^{2}$/s]	$D_{s,pc}$ [cm${}^{2}$/s]	$\frac{D_{s,pc[cycled]}}{D_{s,pc[uncycled]}}$	$\frac{D_{s,pa[cycled]}}{D_{s,pa[uncycled]}}$
Uncycled	3.8211	3.7157	0.1054	$5.10\times 10^{-10}$	$3.48\times 10^{-10}$	0.5	0.4
Cycled	3.8213	3.7107	0.1106	$2.73\times 10^{-10}$	$1.56\times 10^{-10}$		

**Table 5:** Mean and standard deviation of $Li^{+}$ diffusion in NMC uncycled and cycled electrodes.

	NMC			
	Uncycled		Cycled	
Mean and Standard Deviation	Lithiation	Delithiation	Lithiation	Delithiation
Mean [cm${}^{2}$/s]	$3.50\times 10^{-10}$	$5.11\times 10^{-10}$	$1.59\times 10^{-10}$	$2.72\times 10^{-10}$
Standard deviation [cm${}^{2}$/s]	$4.85\times 10^{-12}$	$4.52\times 10^{-12}$	$2.10\times 10^{-12}$	$4.55\times 10^{-12}$

**Table 6:** Diffusion coefficients in uncycled and cycled electrodes at 3.71 V determined by GITT and their diffusivity ratio.

Electrode Type	$D_{s}$ [cm${}^{2}$·s${}^{-1}$]	$\frac{D_{s,[cycled]}}{D_{s,[uncycled]}}$ [/]
Uncycled	$2.98\times 10^{-14}$	2.9
Cycled	$8.60\times 10^{-14}$	

**Table 7:** Diffusion coefficients in uncycled and cycled electrodes at 3.71 V determined by EIS and their diffusivity ratio.

Electrode Type	$D_{s}$ [cm${}^{2}\cdot$s${}^{-1}$]	$\frac{D_{s,[cycled]}}{D_{s,[uncycled]}}$ [/]
Uncycled	$1.93\times 10^{-13}$	0.4
Cycled	$7.14\times 10^{-14}$	

**Table 8:** Ratios in the diffusion coefficients determined by GITT and EIS techniques compared to CV.

Electrode Type	$D_{s,\mathrm{GITT}}/D_{s,\mathrm{CV}}\left[/\right]$	$D_{s,\mathrm{EIS}}/D_{s,\mathrm{CV}}\left[/\right]$
Uncycled	$8.56\times 10^{-5}$	$5.54\times 10^{-4}$
Cycled	$5.51\times 10^{-4}$	$4.57\times 10^{-4}$

## Abbreviations

The following abbreviations are used in this manuscript:

BEV	Battery Electric Vehicle
CC	Constant Current
CV	Cyclic Voltammetry
CV	Constant Voltage
EIS	Electrochemical Impedance Spectroscopy
GITT	Galvanostatic Intermittent Titration Technique
HEV	Hybrid Electric Vehicle
NMC	Nickel Manganese Cobalt
PHEV	Plug-In Hybrid Electric Vehicle
SoC	State of Charge
SoH	State of Health
XRD	X-ray diffraction
